# Relative Impact of Underreporting and Desistance on the Dark Figure of Sexual Recidivism

**DOI:** 10.1002/bsl.2715

**Published:** 2025-01-22

**Authors:** Nicholas Scurich, Richard S. John

**Affiliations:** ^1^ University of California Irvine California USA; ^2^ University of Southern California Los Angeles California USA

**Keywords:** desistance, Monte Carlo simulation, sexual abuse, sexual recidivism, underreporting

## Abstract

Sexual recidivism rates based on arrests or convictions underestimate actual reoffending due to underreporting. A previous Monte Carlo simulation estimated actual recidivism rates under various reporting and conviction assumptions but did not account for desistance—the decreasing likelihood of reoffending over time. This study addresses that gap by incorporating a 12.3% annual desistance rate (from a well‐known empirical study) and exploring its impact alongside varying charge rates (100%–5%). The results showed that reductions in charge rates lead to disproportionately large increases in recidivism. For instance, lowering the charge rate from 50% to 25% results in a much larger increase in actual recidivism than reducing it from 100% to 75%, despite both being 25% reductions. This indicates that as charge rates decrease, actual recidivism grows more sharply. A sensitivity analysis also examined desistance rates of 0%, 5%, 12.3%, and 20%. Higher desistance rates cause reoffending to occur earlier but have little impact on long‐term totals. Over 25 years, reoffending rates remain similar across desistance rates, suggesting desistance affects the timing, but not the overall amount of reoffending.

*All models are wrong, but some are useful*‐‐

George Box (1976).

The research literature on recidivism of sexual offenders is voluminous. Hundreds of empirical studies from all over the world have examined the topic. Although the studies are heterogeneous in many respects, the vast majority of them define sexual recidivism as a new arrest or criminal charge for a sexual offense (Hanson and Bussiere 1998). The studies, thus, rely on official crime statistics (e.g., arrest or conviction) to measure recidivism.

Sexual offenses—like all criminal offenses—are underreported to authorities. As a result, official crime statistics underestimate the actual rate of sexual offending (Lynch 2011; Rice and Harris 2006; Scurich 2020). The difference between offenses that occur and offenses that occur and get reported to authorities is known as “the dark figure of crime” (Biederman and Reiss 1967).

The dark figure of sexual offenses has been observed in multiple studies using a variety of methodologies, including (a) victim self‐report data, (b) offender self‐report data, (c) forensic DNA studies, and (d) probabilistic modeling (See Lussier et al. 2023; Delisi et al. 2016; Lovell et al. 2020; Charette and Vere van Koppen 2016). However, attempts to even roughly estimate the impact of underreporting on sexual recidivism rates are immensely controversial. Indeed, Lussier et al. (2023) noted that such efforts might be considered “taboo, with researchers avoiding the issue altogether because it raises doubts about the current foundations of SOR (sex offender recidivism) research” (p. 16).

An analysis by Scurich and John (2019) addressed the dark figure issue by using a Monte Carlo simulation methodology, which is commonly used in engineering, economics, finance, medicine (Kroese et al. 2014; Mode 2011), and criminology (Hunt and Miles 2015). Monte Carlo simulation relies on repeated random sampling to quantify model uncertainty and give probability estimates of outcomes under different conditions or assumptions. Scurich and John (2019) used a wide range of assumptions in their models to account for the heterogeneity of estimates in the literature and the fact that such parameters will depend on a variety of factors, including jurisdiction and offender population. Their analysis included 36 configurations of different assumptions about victim reporting rates, conviction rates, and the individual offender's propensity to reoffend. The discrepancy between actual versus observed recidivism rates was significant under virtually any configuration of assumptions.

The Scurich and John (2019) article has been discussed extensively by researchers, clinicians, and lawyers. The Pennsylvania Supreme Court discussed it in Commonwealth v. Torsilieri (2020), a case challenging the constitutionality of the sexual offender registry. In Torsilieri (2020), the Pennsylvania Supreme Court remanded the case back to the trial court for an evidentiary hearing to hear expert testimony on the Scurich and John (2019) analysis and other issues related to sexual recidivism and the registry. In 2022, the trial court published an opinion that evaluated the Scurich and John (2019) analysis based on the testimony of several expert witnesses (Commonwealth v. Torsilieri 2022).^1^

The Scurich and John (2019) article engendered several critical responses, both in the academic literature and the expert testimony in Torsilieri (2022). The criticisms of the analysis could be broadly categorized along the following lines: (1) disagreement about empirical assumptions, (2) speculation about differences between convicted and non‐convicted sexual offenders, and (3) that the model failed to account for changes over time. We summarize each of these criticisms below. This article principally aims to address and account for the changes over time criticism.

## 1 Disagreement About Empirical Assumptions

As noted, Scurich and John (2019) used a wide range of assumptions in their models because the sexual offender recidivism research is heterogeneous in the extreme, including, for example, the length of follow‐up, the selection of study participants, and the definition of recidivism (Lussier et al. 2023). A corollary of this heterogeneity was sagely noted by Vern Quinsey almost 40 years ago: “by selectively contemplating the various studies, one can conclude anything one wants about sex offenders' recidivism rates” (cited in Rice and Harris 2006, 95). Critics of Scurich and John (2019) claim that they selectively sampled studies. For example, Lave, Prescott, and Bridges (2021) assert, “Scurich and John tend to rely on studies with higher‐than‐average, unrepresentative recidivism rates and overlook studies that report low recidivism rates” (p. 287).

Scurich and John (2019) relied on four studies to benchmark the observed recidivism rates in sexual offender studies. At one end of the spectrum was Hanson, Steffy, and Gauthier (1993), who reported a 40% recidivism rate over 31 years, and at the other end of the spectrum was Hanson et al. (2014), who reported a 5% recidivism rate in 5 years. They also relied on the Hanson and Bussiere (1998) meta‐analysis, which reported a 15% recidivism rate in 5 years. Thus, the observed recidivism rates used by Scurich and John (2019) ranged from 5% to 40%. A comprehensive review by Lussier et al. (2023) found that across 808 empirical studies, the observed sexual recidivism rate varied from 0% to 68%.

Critics also disputed the assumptions about the victim reporting rates (i.e., 15%, 35%, 100%) and the conviction rates (i.e., 25%, 50%, 75%). These criticisms, however, are notably conflicting. For example, Abbott (2020) stated that the range of conviction rates assumed by Scurich and John (2019) was too high: “Several studies reveal that between 6.5% and 14% of individuals arrested for sexual crimes are later convicted for sexual offenses. This range of values is less than even the lowest limit of 25% [conviction rate] considered in the simulation model” (p. 551, citations omitted). On the other hand, Lave, Prescott, and Bridges (2021) concluded that the opposite was true: “For all of these reasons, the range of values Scurich and John assume for Pc (probability of conviction) is unreasonably low” (p. 294). In other words, Abbott (2020) says a 25% conviction rate is too high, and Lave, Prescott, and Bridges (2021) say a 75% conviction rate is not high enough. Unlike Abbott (2020), Lave, Prescott, and Bridges (2021) cite no empirical evidence whatsoever for their assertion that a 75% conviction rate is “unreasonably low” nor do they specify what conviction rates are reasonable.

Conflicting criticism also occurred concerning the range of assumptions for victim reporting rates (i.e., 15%, 35%, 100%). For example, Abbott (2020) noted wide variation in the reporting rates of children and noted that some of the rates observed in surveys of children were even lower than 15%. Abbott (2020) ultimately concluded the reporting rate for previously convicted sexual offenders was “most consistent” with the 35% assumption (p. 9). On the other hand, Lave, Prescott, and Bridges (2021) refer to the same survey cited by Abbott and point out that “76.1% of sexual abuse by a nonspecified adult and 13.1% of peer sexual abuse is known to the police. These numbers are significantly higher than those Scurich and John report in their article” (p. 291). Lave, Prescott, and Bridges (2021) also cited a study in which 75% of women reported their sexual assault to the police and concluded that “reporting rates may be closer to the ‘optimistic’ [100%] estimate rather than the much lower values pushed by Scurich and John” (p. 292). Of course, Lave, Prescott, and Bridges (2021) failed to acknowledge that the sample in the latter study consisted of individuals *already involved with law enforcement* (see Jones et al. 2009, 421), making these selected data incommensurate with broader population studies.

In the end, Quinsey's four‐decade‐old observation rings true: the extremely heterogeneous nature of the literature enables commentators to selectively credit studies that fit their narrative. Conspicuously absent from critics is an alternative model based on preferred assumptions demonstrating any material difference in the result.

## 2 Hypothesized Differences Between Convicted and Non‐Convicted Sexual Offenders

Another criticism is that convicted and non‐convicted sexual offenders putatively differ in material ways, and these differences impact the results. For example, Helmus (2021) posited, “Scurich and John's (2019) analyses presume that reporting rates, successful prosecution rates, frequency of offending rates, etc., do not meaningfully differ for individuals who have never been caught versus those already known to the system. Their modeling also assumed these factors would be similar across risk levels and offense types (e.g., contact vs. non‐contact offenses), which is unlikely” (p. 15). Similarly, Lave, Prescott, and Bridges (2021) stated, “If victims are systematically more likely to report crimes committed by individuals previously convicted of a sexual offense, then the reporting probabilities Scurich and John use in their simulations are lower than they should be because P(Report|CSO) > P(Report)” (p. 293). In short, critics argue that both reporting and conviction rates would be higher for a previously‐convicted sexual offender than a non‐previously‐convicted sexual offender.

Both of these conjectures—while theoretically possible—are unsupported by evidence. Dr. R Karl Hanson confirmed as much in sworn testimony in the *Torsilieri* hearing:

Q. You indicate in your report that it is likely that individuals with a prior conviction would be more likely to be detected than other individuals. Do you recall saying that in your report?

A. Yes, I do. And I believe that to be true.

Q. And you believe it to be true based on your beliefs, but you don't have research that backs up that claim, correct?

A. That is correct. (Testimony given on June 28, 2021, p. 188)

It is possible that the opposite is true, and previously convicted sexual offenders become better at escaping detection than first‐time offenders. For example, evidence suggests that “successful” sexual offenders get better at avoiding detection as they commit more offenses (Lussier, Bouchard and Beaureguard 2011).

Only one study has addressed this issue empirically. Kelley et al. (2023) examined a sample of 200 men who were committed as sexually violent predators in Wisconsin. This is a retrospective study, looking backward at the criminal histories of these men who are now incarcerated. Kelley et al. (2023) reviewed Department of Corrections and pre‐sentencing reports as well as criminal justice databases to ascertain the criminal history of each man. These men also completed sexual history disclosure surveys and were polygraphed regarding their offense history. Responses to the disclosure survey and polygraph were compared to the criminal justice records to ascertain how many offenses were detected and how many were not detected by law enforcement but were disclosed on the survey or polygraph.

Kelley et al. (2023) define a “detected” offense as one that resulted in a formal sanction, that is, “detained, arrested, or charged” (p. 8). On the other hand,

An undetected victim was defined as a victim of a sexual offense that would have counted towards the Static‐99R scoring had the individual been detected and arrested, charged, or convicted for the offense. The victim/offense may have been “detected” or known by police, social services, or others but was considered undetected if the individual was never formally sanctioned (e.g., detained, arrested, or charged). (p. 8)

To be clear, even if an offense was brought to the attention of authorities, it was not considered “detected” unless the individual was formally sanctioned. This criterion differs from victim self‐report surveys, which ask whether the sexual offense was reported to the police, which alone would not count as “detected” in the Kelley et al. (2023) study.

There were ultimately 189 men included in their analysis. Before their first arrest for a sexual offense, the sample had 1103 victims, of which 253% or 22.6% were “detected”. (Table 2, p. 13). After they were released from custody, there were 641 additional victims, of which 232% or 36.2% were “detected.” The difference in detection percentages (22.6% vs. 36.2%) is statistically significant, suggesting that the detection rate (i.e., arrest or charge) does increase for convicted sexual offenders relative to non‐convicted sexual offenders. However, “this effect only occurred after the first sanction” (Kelley et al. 2023, 1). The detection rates did not continue to increase as offenders were released and reconvicted for additional sexual offenses.

Notwithstanding the acknowledged limitations of this study—namely, potential generalizability issues, including the extraordinarily high recidivism rate: 75% of the sample committed a new detected sexual offense after being released for their first sexual offense—the study does provide some support for the hypothesis that convicted and non‐convicted sexual offenders differ in their detection rates.

How does this difference affect the results reported by Scurich and John (2019)? Although the operational definition of “detected” in the Kelley et al. (2023) study is not the same as the victim report surveys relied upon by Scurich and John (2019) to estimate the “reporting” rate, the value of 35% is identical—meaning that this distinction between convicted and non‐convicted sexual offenders, while theoretically important, has no impact on the estimates provided in the Scurich and John (2019) analysis (cf. Abbott, 9).

## 3 Changes to Recidivism Risk Over Time and “Desistance”

According to the trial court opinion from the Torsilieri (2022) hearing:

As Dr. Hanson testified, there are no findings in that [Scurich & John] study. It is a statistical model based on certain assumptions. If you follow those assumptions, you get that result. I do not agree with the assumptions. They [sic] are two fundamental areas of disagreement.

Their model assumes recidivism risk is a constant that does not change over time. This assumption is not supported by the data. Recidivism does change over time.

They also assume that most individuals who do reoffend do so rarely, once in a while. They also have no category for no recidivism. So they don't create a category of people who do not reoffend, so to speak. (pp. 8–9)

This last criticism—that the model did not include offenders for whom the probability of recidivism is zero—is obtuse. Any living person who is not in a permanent vegetative state has a non‐zero chance of recidivism. Indeed, Hanson recently published a paper entitled, “There is no such thing as zero risk of sexual offending” (Lee, Brankley, and Hanson 2023). The other criticism—that the model assumed a constant rate of offending over time—is at least plausible based on the criminological concept of “desistance.”

The National Institute of Justice defines desistance as “the process by which criminality, or the individual risk for antisocial conduct, declines over the life‐course, generally after adolescence (Rocque 2021).” Some have argued that the risk of sexual recidivism declines over time as an offender remains offense‐free in the community (Hanson 2018). Scurich and John's (2019) models explicitly did not incorporate this feature:

The primary limitation of this probabilistic model is the absence of attention to dependency, sequence, and feedback. Dependencies between uncertainties about the number of offenses committed and the likelihood of each being reported and/or successfully prosecuted are not accounted for in the model; all uncertainties are modeled as independent (hence uncorrelated). Although this assumption is reasonable as a first approximation, accounting for dependencies and estimating their impact can be accomplished within the Monte Carlo simulation by assessing correlations among uncertainties (p. 171).

The current article seeks to understand how changes in risk over time (desistance) and the dark figure impact the actual rate of sexual recidivism.

### 3.1 Conceptualizing Desistance From Sexual Offending

Criminological research on desistance studies the *process* by which offenders abstain from criminal activity instead of a binary termination point (Kazemian 2007). This process includes many dimensions, including changes to cognitions, emotions, decision making, awareness, and behavior. Various measures and data sources have been employed to study desistance, though it is widely recognized that official crime statistics provide an indirect and incomplete measure and that self‐report surveys are a more appropriate measure (Rocque 2021).

Studies of sexual offender desistance rely on official crime statistics and show that the risk of *re‐arrest* decreases the longer the offender remains arrest‐free in the community (e.g., Kruttschnitt, Uggen, and Shelton 2000). The most well‐known study was conducted by Hanson et al. (2018), which combined 20 different samples of sexual offenders that were tracked for up to 25 years. A logistic regression was conducted every 6 months to predict the chances of being re‐arrested. Hanson et al. (2018) reported that “each year offense‐free was associated with a 12% decrease in the odds of recidivism (e[−0.131] = 0.877)” (p. 54). Hanson et al. (2018) concluded that “Within 10–15 years, the vast majority of individuals with a history of sexual crime will be no more likely to commit a sexual crime than individuals who have been convicted of a non‐sexual crime and who have never been previously convicted of a sexual crime (1%–2% after 5 years; Kahn et al. 2017)” (p. 57).

Criminologists have criticized both this conceptualization and the approach to measuring desistance (Cooley 2022). For example, Cooley and Sample (2018) noted that “Desistance includes gradual cognitive and affective transformation, whereas the lack of reoffending could be a function of several factors, including the natural aging process, the lack of opportunity, or increases in social bonds” (p. 483). Furthermore, they note:

[I]t is intellectually irresponsible of us to continue to run regression models to predict a concept we are not measuring. Models to understand desistance from sex offending should include thoughts, emotions, identity traits, and feelings that change as social circumstances do. Without the measure of psychological transformations among offenders, we are left with finding attributes that are associated with a lack of reoffending, not desistance. To improve sex offender policy, the term ‘desistance’ should no longer be bandied about and inferred from studies on sexual recidivism and a lack of reoffending. It is time to further explore the thoughts, decisions, and emotions associated with the concept of desistance as a process rather than simply calling offenders ‘desisters’ if they have not reoffended. (p. 498)

We do not wish to enter the debate on how to conceptualize and measure desistance from sexual offending. We only note that Hanson et al.'s (2018) approach is controversial and may not be measuring what it claims. Nevertheless, given the prominence of the analysis, we accept this approach for the purposes of this paper.

## 4 Modeling the Impact of Desistance on Actual Sexual Recidivism Rates

### 4.1 The Basic Model

This section presents an extension of the Monte Carlo simulation model proposed by Scurich and John (2019) to estimate the actual recidivism rate of sexual offenders. The original model assumed that reoffending followed a Poisson distribution for the period of interest. A Poisson distribution is typically used to model rare events (e.g., crimes committed over time, Maltz 1996; Caulkins 2001; Rhodes, Kling, and Johnston 2007). In that model, each individual's reoffending risk was characterized by their Poisson mean, which remained constant for the period of interest. The offender population distribution of reoffending risk (Poisson means) was assumed to follow an Exponential distribution. An exponential distribution is highly skewed, meaning most offenders have a low reoffending risk, while a few have a much higher risk (DeGroot 1970).^2^

The mean of the Exponential distribution was selected to match the empirical reoffending rate reported in four different studies, ranging from 5 to 31 years, for various combinations of the probability offenses are reported and the probability that reported offenses are successfully prosecuted. The simulation analysis results indicated that actual reoffending rates were substantially greater than the observed reoffending rates reported. For example, the actual 25‐year reoffending rate was estimated to range between 32% (100% reporting, 75% successful prosecution) and 90% (15% reporting, 25% successful prosecution), based on the observed 25% conviction rate over 25 years reported by Prentky et al. (1997).

Using a dataset aggregated from 20 samples (*N* = 7225 individuals, *N* = 105,347 6‐month observation periods), Hanson et al. (2018) constructed a binary logistic regression model indicating that the odds of reoffending decreased by 12.3% annually over 25 years. In the current extended simulation model, we assume that the mean of the Poisson distribution for the annual number of offenses decreases such that the odds of reoffending decrease by 12.3% each year. The Year‐1 Poisson mean (*λ*_1_) is randomly chosen from an Exponential distribution with mean *μ*. Following Scurich and John (2019), *μ* is selected to define a population of offenders that produce the 25‐year reoffending rate reported by Hanson et al. (2018), 18.5%. The Poisson mean number of offenses in Year *i* + 1, *λ*_*i*_ _+_ _1_, is calculated from *λ*_*i*_:

(1) $$\lambda_{i+1}=\ln\left(1+0.877*\left(1-\exp\left(-\lambda_{i}\right)/\exp\left(-\lambda_{i}\right)\right)\right)$$

Equation (1) defines a new Poisson distribution for each year such that the odds of reoffending decreases by 12.3% each year, the core finding from Hanson et al. 2018. (See Appendix A for a proof.).

The current model defines a population of offenders, characterized by the Exponential distribution mean (*μ*), for their starting Year‐1 Poisson annual mean number of offenses. We utilized the Goal Seek option in @Risk, a Monte Carlo simulation Excel add‐in (see Guerrero and Guerrero 2019), to estimate values of *μ* that would produce the same 25‐year reoffending rate reported in Hanson et al. (2018), 18.5%. If there is no dark figure (i.e., 100% of offenses are detected), *μ*_100_ was estimated to be 0.0287, based on 30,000 iterations.

For Year 1, the simulation model draws a value of the Poisson mean for the number of sexual offenses in Year 1, *λ*_1_, from the Exponential distribution with mean *μ*_*r*_, where *r* is the charge rate of sexual offenses. A Binomial distribution, *B*(*n*,*p*), is used each year to model the number of sexual offenses charged, where *n* = the number of offenses from the Poisson and *p* = the probability that a sexual offense will result in charges (Hanson and Howard 2010; Scurich and John 2012). If an offense occurs (as modeled by the Poisson) and the offense is charged (as modeled by the Binomial), the trial is recorded as a reoffense for that year, and the simulation moves to the next iteration. Each year, the Poisson mean, *λ*_*i*_, is adjusted downward using Equation (1), such that the odds of reoffending each year decrease by 12.3%.

### 4.2 The Basic Model Extended to Account for the Dark Figure

The simulation result above (*μ* = 0.0287) assumes that all sexual offenses are charged and result in successful convictions. Of course, as acknowledged by Hanson et al. (2018), “…observed rates underestimate the real recidivism rates because not all sexual offenses are reported and available in the databases used by researchers” (p. 49). Unlike Scurich and John's original model, which included separate uncertainties for reporting a sexual offense and conviction, conditional on a reported offense, the current model focuses on whether a sexual offense results in a charge or conviction (hereinafter “charged”), setting aside whether the offense is reported, police decide to investigate and arrest the suspect, a prosecutor decides to file charges for a sexual offense, and the defendant pleads guilty to a sexual offense or a judge or jury convicts a defendant of a sexual offense.^3^ Accordingly, this model examines the impact of different charge rates on actual sexual recidivism. Six charge rates (*r*) were selected: *r* = 75%, 50%, 35%, 25%, 15%, and 5%.

**FIGURE 1:**
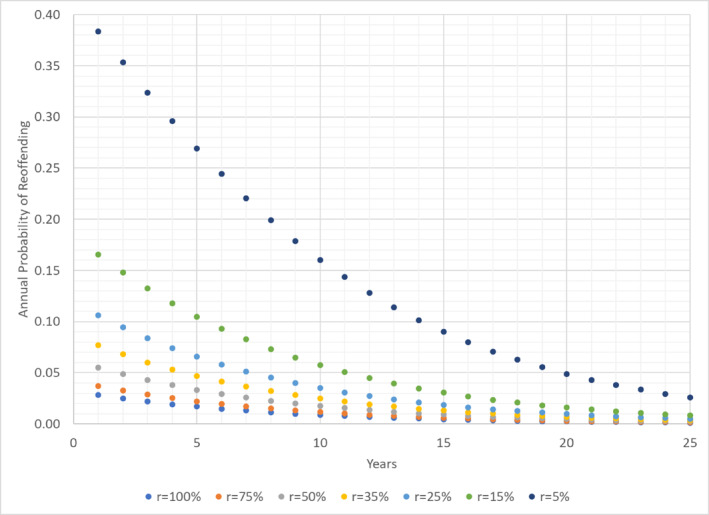
Annual probability of reoffending each year by simulated charge rate (*r*).

**FIGURE B1:**
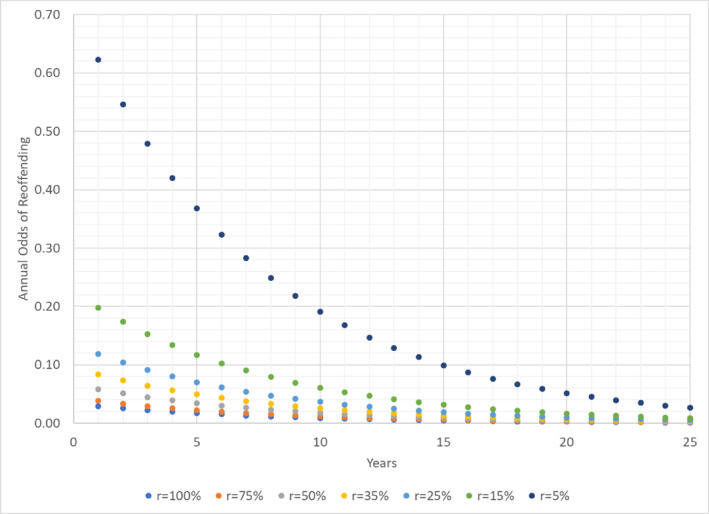
Annual odds of reoffending by simulated charge rate (*r*). Odds for Year_*i*_ _+_ _1_ = 0.877 × Odds for Year_*i*_.

**FIGURE B2:**
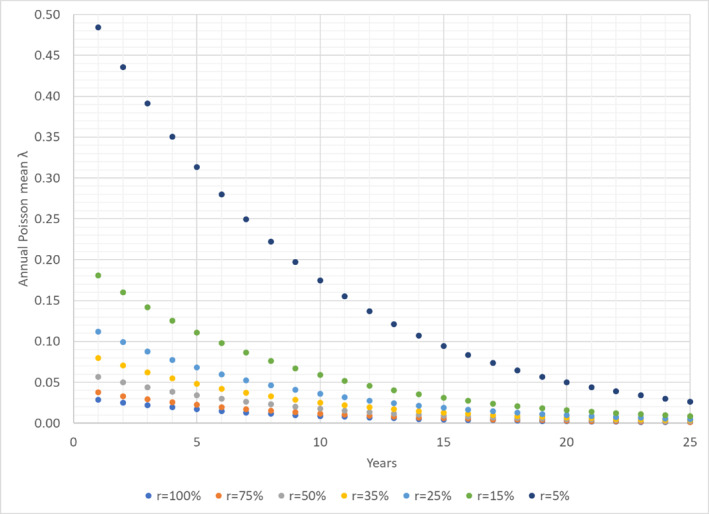
Annual poisson mean number of reoffenses (*λ*_*i*_) by simulated charge rate (*r*). *λ*_*i*_ _+_ _1_ defined in Equation (1).

Figure 1 plots the annual probability of reoffending for each of the six charge rates considered (75%, 50%, 35%, 25%, 15%, and 5%) and the base‐case of 100% charged, setting *λ*_1_ = *μ*_*r*_, over 25‐years. These plots all indicate a dramatic decrease in reoffending rates over time, corresponding to the empirical results from the 20 convenience samples reported by Hanson et al. (2018). All values of *μ*_*r*_ were estimated using the Goal Seek function in @Risk so that the 25‐year charge rate was held constant at 18.5%. These values of *μ*_*r*_ must increase as the Binomial probability of a sexual offense charge (or conviction), *r*, decreases. The 100% charge rate plot (medium blue dots at the bottom) corresponds to the plot of green dots in Figure 1 by Hanson et al. (2018). We also plot the annual odds of reoffending (Figure B1 in Appendix B) and the annual Poisson mean number of offenses, *λ*_*i*_ (Figure B2 in Appendix B), over 25 years for each of the six charge rates and the base‐case (100% charged).

Table 1 presents estimates of the actual 5, 10, 15, 20, and 25‐year reoffending rates for each of the six charge rates considered (75%, 50%, 35%, 25%, 15%, and 5%) based on 30,000 Monte Carlo simulation iterations. Column 1 lists the values of *μ*_*r*_ based on the Goal Seek analysis described above and used to generate the mean Poisson plots over time (Figure 1). The base‐case charge rate model (*r* = 100%, 2nd row) produces quite similar reoffending rate estimates to those reported by Hanson et al. (2018) (1st row) for all five‐time points. The bottom six rows report actual reoffending rate estimates when the charge rate is less than 100%. The table shows how lower charge rates (*r* < 100%) lead to higher actual recidivism rates. For instance, for *r* = 75%, the 25‐year recidivism rate is estimated at 22.8%, while for *r* = 5%, the rate is 80.0%.

**TABLE 1:** 5, 10, 15, 20, and 25 Year actual reoffending percentages by proportion charged (*r*).

		Years				
*μ*_*r*_	*r*	5	10	15	20	25
Hanson	Estimates	9.1%	13.3%	16.2%	18.2%	18.5%
0.0287	1.00	10.2%	14.7%	16.8%	17.8%	18.3%
0.0376	0.75	12.9%	18.4%	21.0%	22.2%	22.8%
0.0567	0.50	18.1%	25.1%	28.4%	30.0%	30.9%
0.0800	0.35	24.0%	32.6%	36.3%	38.2%	39.1%
0.1123	0.25	30.9%	40.7%	44.7%	46.7%	47.6%
0.1808	0.15	41.9%	52.5%	56.7%	58.7%	59.6%
0.4841	0.05	65.9%	74.9%	77.9%	79.3%	80.0%

*Note: μ*_*r*_ = Exponential mean of the first year Poisson rate of reoffending, *λ*_1_ and *r* = charge rate for each offense.

Table 1 indicates that as the charge rate decreases, the increase in reoffending rates becomes non‐linear. This result indicates that a relatively small reduction in the charge rate leads to a much larger increase in actual recidivism. For example, a charge rate of 50% results in a 25‐year reoffending rate of 30.9%, while a charge rate of 5% results in a rate of 80.0%. Hence, as fewer offenses are charged, more offenders reoffend without detection, leading to a much higher overall reoffending rate. The non‐linear increase in actual recidivism across different charge rates is an important and non‐obvious insight, and it illustrates the importance of considering the charge rate when evaluating recidivism rates, even after accounting for the estimated desistance reported by Hanson et al. 2018 (12.3% decrease in the odds of reoffending).

### 4.3 The Basic Model Extended to Account for the Dark Figure and Varying Rates of Desistance

The desistance rate reported by Hanson et al. (2018) of 12.3% comes from a convenience sample of studies. The studies vary widely along methodological and geographic lines. For example, only four of the studies are from the United States, and only one of those four is published in a peer‐reviewed journal. Given differences in observed recidivism rates across countries (Lussier and McCuish 2024), it is reasonable to question the generalizability of the 12.3% annual desistance rate. Therefore, we conducted an additional sensitivity analysis that varied the 12.3% desistance rate.

We repeated the analysis reported above for annual decreases in odds of reoffending of 20% (a faster decrease in reoffending risk), 5%, and the limiting case of 0% annual decrease in odds, used by Scurich and John (2019). We used the same Goal Seek approach to estimate *μ*_*r*_ for all six values of charge rate (*r*) and the base case of *r* = 100%. In all cases, *μ*_*r*_ values were estimated to match the 18.5% reoffending rate at 25‐years estimated by Hanson et al. (2018).

Table 2 presents 5‐ and 25‐year actual reoffending percentages by the proportion charged rate (100%, 75%, 50%, 35%, 25%, 15%, 5%) and annual desistance rates (20%, 12.3%, 5%, and 0%). The rows for an annual 12.3% decrease in the odds of reoffending correspond to the analysis reported above and are included for comparison purposes. The rows of 0% desistance are comparable to the results of the original model reported by Scurich and John (2019).

**TABLE 2:** 5‐ and 25‐year estimated actual reoffending percentages by proportion charged (*r*) and annual desistance rate (decrease in odds of reoffending).

5‐Year							
Proportion charged							
Annual desistance rate	1.00	0.75	0.50	0.35	0.25	0.15	0.05
20%	13.2%	16.9%	23.0%	30.7%	37.5%	48.7%	71.4%
12.3%	10.2%	12.9%	18.1%	24.0%	30.9%	41.9%	65.9%
5%	6.5%	8.5%	12.4%	17.2%	21.8%	31.5%	57.7%
0%	4.3%	5.6%	8.3%	11.3%	15.2%	23.4%	47.7%

25‐Year							
Annual desistance rate	1.00	0.75	0.50	0.35	0.25	0.15	0.05
20%	18.5%	23.4%	31.1%	39.8%	47.2%	58.6%	78.9%
12.3%	18.3%	22.8%	30.9%	39.1%	47.6%	59.6%	80.0%
5%	18.3%	23.0%	30.9%	39.1%	46.7%	59.5%	81.6%
0%	18.6%	23.1%	31.7%	39.4%	47.8%	60.7%	82.1%

The actual reoffending percentages at 5 years (top panel) are inversely influenced by the charge rate (*r*), regardless of the desistance rate. In the short term (5 years), higher desistance rates (e.g., 20%) lead to higher reoffending rates compared to lower desistance rates (e.g., 0%). This result indicates that more reoffending occurs early in the time frame when the odds of reoffending decrease faster. For example, at a 100% charge rate with a 20% desistance rate, the 5‐year reoffending rate is 13.2%; with a 0% desistance rate, it's 4.3%.

The reoffending rates at 25 years (bottom panel) indicate the same inverse relationship with the charge rate; the lower the charge rate, the greater the actual reoffending rate for all four annual desistance assumptions. However, over 25 years, the actual reoffending rates are nearly identical across different desistance rates for each charge rate. For example, at a 100% charge rate with a 20% desistance rate, the 25‐year reoffending rate is 18.5%; with a 0% desistance rate, it's 18.6%. The difference is negligible. With a 5% charge rate and a 20% desistance rate, the 25‐year reoffending rate is 78.9%, while at a 0% desistance rate, it's 82.1%. When the charge rate is very low (5%), the desistance rate has little long‐term impact, though the difference is slightly more noticeable. However, the overall reoffending rate remains very high due to the low charge rate.

This sensitivity analysis suggests that desistance mainly affects when reoffending happens (earlier or spread out over time) but it does not have much impact on the total amount of reoffending over the long term. The model spreads reoffending rates more evenly across the period for no or low (5%) annual desistance. In contrast, the model places most reoffending in the first 5 years when desistance rates are larger (12.3% or 20%). The charge rate is the dominant factor influencing actual reoffending rates, especially in the long term. Lower charge rates lead to much higher actual reoffending rates because fewer offenders are caught and charged.

## 5 Discussion

### 5.1 Summary of Findings

We have presented an extended model of that proposed by Scurich and John (2019). The model accounts for desistance, or the decrease in the risk of reoffending over time. We calibrated the model to match the 18.5% 25‐year reoffending rate and the 12.3% annual decrease in odds of reoffending estimated from 20 datasets by Hanson et al. (2018). We demonstrate that the model assumptions can reproduce the reoffending rates at 5, 10, 15, 20, and 25 years reported by Hanson et al. (2018), assuming 100% reporting. We find it remarkable that the Exponential population model for mean Year‐1 reoffending frequency (*λ*_1_) closely approximates the estimated reoffending rates reported in Hanson et al. (2018) (9.1%, 13.3%, 16.2%, 18.2%, and 18.5%), given their aggregation of 20 convenience samples using varying inclusion and exclusion criteria. The aggregated samples analyzed in Hanson et al. (2018) are by no means a random or representative sample from any defined population of sex offenders. Yet our Exponential‐Poisson model of reoffending was a good approximation of the reoffending rates estimated by Hanson et al. (2018) from the combined 20 samples.

Our analysis indicates that empirical estimates of recidivism rates based on sexual offenses charged or successfully prosecuted may be greatly underestimated. The actual 25‐year reoffending rate is over double for the 35% charged rate, over triple for the 15% charged rate, and over quadruple for the 5% charged rate. The sensitivity analysis varying reporting rate provides a useful guide for estimating the actual recidivism rate, conditional on the estimated rate of charged offenses. The analysis presented indicates that actual reoffending rates are highly dependent on the rate at which sexual offenses are charged, even when accounting for desistance.

As a point of reference, one study from Los Angeles found that approximately 12% of rapes or attempted rapes in 2005–2009 resulted in an arrest (Spohn and Tellis 2012), and a study examining data from 15 years in Australia, Canada, England and Wales, Scotland, and the United States found that 14% of sexual violence victims reported the offense to the police, and of those about 30% resulted in an arrest and even fewer resulted in a conviction (Daly and Bouhours 2010). This latter study illustrates the profound impact the operational definition of recidivism (charge vs. arrest) can have on the recidivism rate (see Tyre, Allen, and Pflugradt 2024). Our model used “charged” as a key uncertainty, consistent with Hanson et al. 2018, but it should be recognized that this is an oversimplification that could be addressed by more complicated models that account for different reporting rates, charge rates, conviction rates, etc.

Our sensitivity analysis of desistance (annual decrease in the odds of reoffending) suggests little or no impact of desistance on actual reoffending rates over a long period. Rather, greater annual decreases in the odds of reoffending result in a redistribution of offending such that reoffending occurs sooner than later, conditional on a fixed 25‐year rate of charged sexual reoffending. While the desistance rate gradually reduces the likelihood of reoffending over time, it has a more moderate effect than the charge rate. It ensures that the longer an offender goes without reoffending, the less likely they are to reoffend in the future. However, this effect is cumulative and more gradual over time, unlike the sharp, immediate impact of changes in the charge rate. The simulation shows that the recidivism rate increases dramatically as the charge rate decreases. Even a small drop in the charge rate leads to a substantial rise in reoffending because a lower charge rate means fewer offenders are prosecuted, and therefore, more offenders remain undetected and able to reoffend.

### 5.2 Anchoring and Adjustment

Exactly half a century ago, Tversky and Kahneman published a paper in *Science* on three general classes of heuristics and biases that can potentially cause systematic and predictable errors in judgment. One of the classes of biases identified and described in their paper was “adjustment from an anchor, which is usually employed in numerical prediction when a relevant value is available” (Tversky and Kahneman 1974, 1131). Tversky and Kahneman (1974) provide data suggesting that “adjustments are typically insufficient … biased toward the initial values” (p. 1128). Empirical estimates of sexual reoffending defined by charged offenses are clearly relevant to the problem of estimating actual sexual reoffending rates. However, trying to adjust a reported reoffending rate of 18.5% over 25 years for sexual offenses that are not reported, not investigated, not charged, or not successfully prosecuted is likely to result in systematic underestimation of the actual reoffending rate.

The objective of the original Scurich and John (2019) model and the current extension of the model is to provide insight into the actual sexual reoffending rate. Our model decomposes the estimation problem and provides actual reoffending estimates for various assumptions about the charge rate. Our model is analogous to a cashier at a store calculating the total price of multiple items in the cart. We would not expect the cashier to inspect a few items in the cart and estimate the total by adjusting the price of the items inspected. Likewise, we wouldn't expect the cashier to use heuristics, such as counting the number of items in the cart and multiplying by the price of one item at the top of the cart. Such heuristics would undoubtedly result in systematic and unacceptable errors. Likewise, the use of an empirical estimate of sexual reoffending (18.5%) that must be subjectively adjusted to account for uncharged sexual offenses is likely to result in systematic and unacceptable errors.

We have provided a sensitivity analysis that allows for a range of assumptions regarding the charge rate of sexual offenses. Others are welcome to suggest competing models to estimate actual sexual reoffending rates that account for sexual offenses that are not reported, investigated, charged, or successfully prosecuted. Our objective is to provide a framework for estimating actual sexual offending rates that allows for diverse assumptions. This model is analogous to a calculator that can be used to estimate actual sexual reoffending rates, informed from empirical studies of sexual reoffending that has been charged or successfully prosecuted.

### 5.3 Model Rationale and Limitations

Scurich and John (2019) motivated the proposed Exponential‐Poisson‐Binomial (EPB) model in some detail. We highlight some of the rationale for the model without repeating the information already provided by Scurich and John (2019). The heart of the model is an assumption that a Poisson distribution can represent the frequency of sexual reoffending in 1 year. A gentle and intuitive motivation for using the Poisson distribution to model sexual offending frequency is provided by Scurich and John (2019). It is important to understand that the Poisson is highly right‐skewed, with the greatest probability of no offenses in any given year for the values of *λ* utilized in the model. The Poisson distribution has been used to model criminal offense frequencies for the past half century (see Maltz 1996). The Poisson distribution has also proven to be useful for modeling many different events (Winkler 1972) and is used not only in criminology modeling but in a variety of application areas in operations research (Ross 2014).

There are a few limitations of the model that should be recognized. As noted above, the mean annual rate is adjusted each year such that the odds of reoffending each year decrease by a fixed percentage, e.g., 12.3%. A more general model is also available, allowing for specified adjustments to *λ* over time. While we were prepared to use the more general Gamma distribution, for which the Exponential is a special case, we found that the Exponential provided a reasonable approximation to the Hanson et al. (2018) estimates from the 20 aggregated samples. One direction for future research is to fit a more general distribution, such as a Gamma, to data that are genuinely representative of some population of sexual offenders. Further, The Poisson assumption may underestimate periods of concentrated offending (e.g., some offenders may commit multiple offenses in a short period) or fail to capture complex cycles of offending behavior that don't align with a simple Poisson process.

The model assumes that all offenders follow the same Exponential distribution for their risk of reoffending, with most having low recidivism risk and a few having very high risk. However, in reality, offenders are a heterogeneous group, with different factors (e.g., age, criminal history, type of offense) influencing their likelihood of reoffending. This simplification may obscure important differences between subgroups of offenders, leading to less accurate predictions for high‐risk or low‐risk individuals. For example, offenders with specific psychological or social characteristics may have different desistance trajectories than the model assumes. This complexity motivated our sensitivity analysis with varying desistance rates.

The desistance rate in the model assumes a fixed annual decrease in the odds of reoffending (e.g., 12.3%, 20%, or 5%), which may not fully capture the complexities of how offenders' behavior changes over time. In reality, desistance may not follow a simple, consistent decline, and therefore, the use of a constant desistance rate may oversimplify the dynamics of recidivism (see Harris 2021). For example, some offenders might have fluctuating or context‐dependent reoffending risks, while others may reoffend much later than predicted by the model. The criticism applies equally to commentators who advocate the 12.3% desistance rate in policy discussions.

Finally, the models rely on assumptions about the proportion of offenses reported and successfully prosecuted, which vary across regions and contexts. The charge rates (*r*) used in the models are estimates, and these values may not reflect actual reporting or prosecution rates in practice. Of course, if these assumptions are inaccurate, the estimated recidivism rates may either overestimate or underestimate the actual reoffending rates. Hence, we used a wide range of charge rates from 5% to 100%. Commentators can question any particular charge rate assumption or even speculate about the rate in a given jurisdiction without undermining the model's results.

## 6 Conclusion

The Scurich and John (2019) analysis has faced significant critique from various commentators, which is unsurprising given its central role in the *Torsilieri* matter. Such scrutiny is a hallmark of scientific progress as the field advances through rigorous examination of its underlying assumptions and methodologies. However, dismissing certain scientific inquiries as inherently unknowable due to complexity (Helmus 2021) or assuming “Judges and policy‐makers already understand the basic idea that ‘observed rates’ do not capture all instances of recidivism” (Lave et al. 2021, 299) is anti‐scientific. Real science demands a commitment to answering difficult questions, not a retreat into defeatism or unsupported assumptions. To advance both knowledge and informed decision‐making, we must prioritize rigorous investigation over convenient narratives.

## Author Contributions

N.S. and R.S.J both conceptualized, wrote, and analyzed the data reported in this manuscript.

## Ethical Statement

This article reports synthetic (simulated) data. No human subjects IRB approval is required.

## Conflicts of Interest

The authors declare no conflicts of interest.

## Data Availability

The output of the simulations is available upon request.

## Endnotes

^1^
Neither Scurich nor John served as expert witnesses in this case.

^2^
The Exponential distribution with mean *μ* is a special case of a Gamma distribution with parameters (1, *μ*). The Poisson distribution with a Gamma distributed mean (otherwise known as a Gamma‐Poisson mixture) is equivalent to a Negative‐Binomial distribution.

^3^
We note that this is the same operational definition of recidivism used in Hanson et al. 2018.

## Appendix A The New Poisson Distribution for Each Year Such That the Odds of Reoffending Decreases by 12.3% Each Year, the Core Finding From Hanson et al. (2018)

$$P\left(k\;\text{offenses}\;\text{in}\;\text{year}\right)=\lambda^{k}*\exp\left(-\lambda \right)/k!$$

where lambda is the mean number of offenses for the year.

$$P\left(\text{reoffending}\right)=P\left(1\;\text{or}\;\text{more}\;\text{offenses}\right)=1–P\left(k=0\right)=1–\lambda^{0}*\exp\left(-\lambda \right)/0!=1-\exp\left(-\lambda \right).$$

Oddsofreoffending=1−exp(−λ)/(1+(1−exp(−λ)))=(1−exp(−λ))/exp(−λ).

$$\text{Odds}\;\text{of}\;\text{reoffending}\;\text{in}\;\text{Year}\;i+1=\left(1-\exp\left(-\lambda_{i+1}\right)\right)/\exp\left(-\lambda_{i+1}\right)=0.877*\text{odds}\;\text{of}\;\text{reoffending}\;\text{in}\;\text{Year}\;i=\left(1-\exp\left(-\lambda_{i}\right)\right)/\exp\left(-\lambda_{i}\right)$$

$$\text{Rearranging}\;\text{terms},\text{we}\;\text{have}\;\lambda_{i+1}=\ln\left(1+0.877*\left(1-\exp\left(-\lambda_{i}\right)\right)/\exp\left(-\lambda_{i}\right)\right).$$

## Appendix B The Annual Odds of Reoffending and the Annual Poisson Mean Number of Offenses, λi Are Plotted Over 25 Years for Each of the Six Charge Rates and the Base‐case (100% Charged)

See Figure B1.

See Figure B2.
